# Estimating Method of Maximum Infiltration Depth and Soil Water Supply

**DOI:** 10.1038/s41598-020-66859-0

**Published:** 2020-06-16

**Authors:** Zhongsheng Guo

**Affiliations:** Institute of Soil and Water Conservation, Northwestern A & F University, Chinese Academy of Science, 26 Xinong Road, Yangling, Shaanxi Province 712100 P. R. China

**Keywords:** Ecology, Hydrology

## Abstract

The maximum infiltration depth and soil water supply must be evaluated in order to estimate the soil water resource use limit by plants and soil water carrying capacity for vegetation, and realize the sustainable use of soil water resources. However, there is no non-destructive method to estimate maximum infiltration depth and soil water supply. We conducted a simulated infiltration experiment and a long-term fixed-position investigation *in situ* in artificial Caragana shrubland at the Guyuan Eco-experimental Station in the semiarid Loess Plateau. The results showed that infiltration depth for one rain event was equal to the distance from the surface to the crossover point between the two soil water distribution curves with soil depth before a rain event and after the rain event. The soil water supply for one rainfall event was the difference in the soil water resources in the soil layers from maximum infiltration depth that occurred after a long period, and could be estimated by a series of two-curve methods. A maximum infiltration depth of 2.9 m occurred in the artificial Caragana shrubland. The results provide a foundation for controlling soil degradation and sustainable use of soil water resources in water-limited regions.

## Introduction

Infiltration is a general hydrological phenomenon of water movement in porous media and the hydrological phenomenon. Water infiltration is highly dependent on two soil factors: soil water conductivity and soil absorptivity^1,2^. Rainfall from the canopy can infiltrate into the soil, known as the infiltration process, or run off along the slope when rainfall intensity exceeds the infiltration rate^3,4^. Some of the water that infiltrates into soil can be stored in soil for plants to utilize or passes through the soil layer and replenishes groundwater.

The Loess Plateau in northern China is a unique geographical unit. Soil layers formed on the Loess Plateau are in the depth range of 30–80 m from the surface^4,5^, and the water table is deep^6^. Irrigation is not practiced in most of the region and so rainwater is the primary source of soil water for artificial woodland or shrubland in the region^4^.

Soil erosion is a worldwide land degradation process and a serious threat to sustainability of agriculture^7^. China is among the countries that suffer from the most serious soil erosion. For example, more than 1.6 billion tons of sediment from the Loess Plateau are dumped into the Yellow River every year^8^.

Vegetation measures and soil management practices are of vital importance in preventing soil erosion in agricultural lands^7^. Over the last 60 years, large areas have been planted with trees and grasses to reduce runoff and soil loss because of their effectiveness. As a result, sediment runoff has reduced from 1.6 billion ton per year in the 1970s to 0.31 billion tonnes per year in recent years and runoff has halved on the Loess Plateau. However, the soil water conditions for plants in most of this region have deteriorated^9–12^. As a consequence, soil becomes desiccated, leading to soil degradation, vegetation decline and eventually desertification in the artificial perennial grassland and woodland areas of the Loess Plateau^10–12^. This, in turn, affects soil quality, artificial vegetation growth and its ecological benefits.

The plant–water relationship can be improved by reducing stand density based on the soil water-carrying capacity for vegetation (SWCCV) when the soil water resources within the maximum infiltration depth (MID) equal the soil water resource use limit by plants (SWRULP). The SWRULP is defined as the soil water resources in the MID when the soil water content within the MID equals the wilting point of an indicator plant^13^. The SWCCV is the maximum amount of indicator plants in a plant population that soil water resources of a unit area can sustain and allow to grow healthily in a given period and place^12^. An indicator plant is the constructive species for natural vegetation or principal or purpose species of trees or grasses of a non-native plantation^4^. The dynamic relationship between plant growth and soil moisture in the root zone has become a key issue in the study of plant ecological water requirements^14^.

Different types of instruments are used to measure infiltration rate. The most common is the double-ring infiltrometer^15^, which was used by Moroke to measure the infiltration rate under different tillage systems in eastern Botswana and other field conditions^16,17^. Another device is the flume infiltrometer^18^. Kato measured infiltration rates according to simulated rainfall; however, it is difficult to determine MID using this method^19^.

Mathematical models are among the most useful tools for studying infiltration. In addition to the infiltration models^20^, Thony studied the relationship between electrical potential gradients and soil water flux under natural fallow. Measuring soil moisture diffusivity with the horizontal soil column method^21^, Zhou developed a mathematical model of infiltration^22^. These mathematical models can be used to evaluate the cumulative infiltration amount (soil water supply)^23^.

In view of the importance of infiltration in soil water management, infiltration depth is determined by the wet–dry boundary identified by digging in the soil^24^. However, this method is unsuitable for continuous measuring in undisturbed soil such as forests. Li *et al*. studied the changes of wetting front in disturbed soil over time in the process of infiltration using a large lysimeter with a long access pipe^25^. In addition, Li & Shao analyzed the influences of vegetation cover, rainfall intensity and soil texture on infiltration rate^26^. Wang & Li determined the MID using an oven drying method^27^. Esteves *et al*. reported the soil moisture profile at the center of a runoff plot to illustrate the change of infiltration depth with time and compared the observed versus computed values of infiltration depth at the neutron probe access tube^28^; Li reported MIDs in the range of 1–3 m in woodlands of the Loess Plateau^10^.

In recent years, several laboratory experiments on infiltration have been reported, but very little information is available on the method of estimating the MID and soil water supply *in situ* in a nondestructive way in the Loess Plateau region^24,29,30^. In addition, the measurement methods developed in the laboratory cannot be used in field conditions, and disturbed soil not adequately representing undisturbed soil^3,31,32^. Esteves *et al*. and Oyonarte *et al*. estimated infiltration depth using a neutron probe to measure the soil water change with soil depth^28,33^. There is no universally accepted method for estimating infiltration depth and soil water supply of natural soil. Therefore, it is necessary to develop a new method for estimating infiltration depth and soil water supply under field conditions to better understand the relationship between plant growth and soil water and to determine soil water resources, SWRULP and SWCCV^4^. This study presents the two-curve method for estimating infiltration depth and soil water supply of natural soil.

## Material and Methods

### Site description

**Figure 1:**
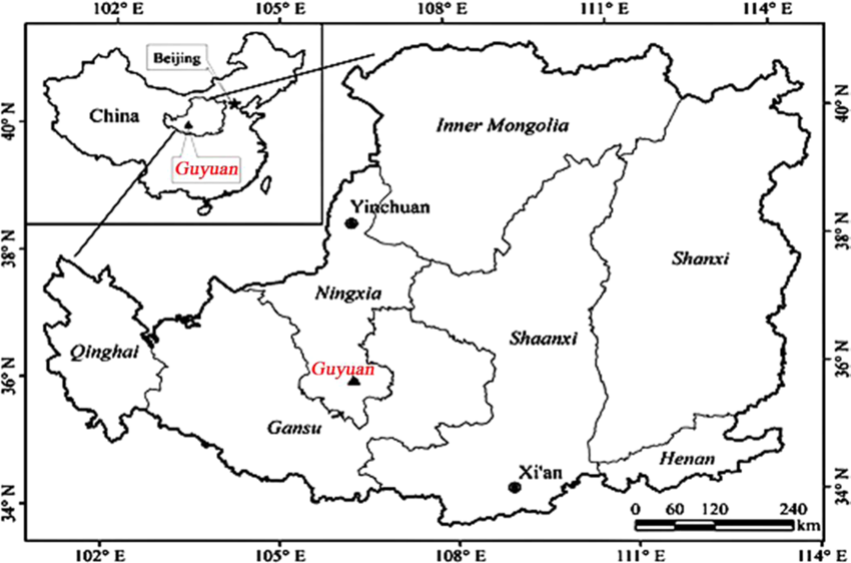
The location of the study site on the loess plateau of China sowed in 2002.

This study was conducted at the Shanghuang Eco-experiment Station operated by the Institute of Soil and Water Conservation, Chinese Academy of Sciences, located in the semiarid region of the Loess Plateau (35°59′–36°02′N, 106°26′–106°30′E) in Guyuan, Ningxia Hui Autonomous Region^34^, see Fig. 1. The altitude ranges from 1534–1824 m. Precipitation is scarce during January–March, and rainfall for June–September accounts for more than 70% of the average annual total precipitation. Mean annual rainfall during 1983–2001 was 415.6 mm. The frost-free period is 152 days. The soil is a Huangmian soil, which is mainly loamy loess^35^.

*Caragana korshinskii* Kom. has been widely planted in this region to control both desertification and soil erosion^12^. The experimental field is located in 16-year-old Caragana shrubland in the middle of the Heici Mountains, with a slope gradient range of 0–15° in the Shanghuang Eco-experiment Station. The main plant species are *Stipa bungeana* Trin., *Heteropappus attaicus* (Wild) Novpkr.and so on.

### Variables and determination methods

The experiment included a long-term, fixed-position and a simulated rainfall experiment in artificial Caragana shrubland.

Precipitation was measured with standard rain gauges at the study site, which is approximately 50 m from the station. The soil moisture content, plant root distribution and other plant growth parameters were determined.

The long-term, fixed-position experimental plot was located on a gentle slope facing southeast with a gradient of approximately 8°. The west side of runoff plot is uphill. Three sides of the plots were bordered by cement boards of 50 cm in height. A pit (1 m × 1 m × 4 m deep) was dug nearby the experimental site for sampling purposes. A hole was dug around the base of a Caragana shrub and the root distribution investigated^12^. Undisturbed and disturbed soil were sampled from depths of 0–5, 20–25, 40–45, 80–85, 120–125, 160–165, 200–205, 240–245, 280–285, 320–325, 360–365 and 390–395 cm, respectively. The two core samples at every soil depth were collected with cutting rings for measuring soil bulk density, capillary porosity and non-capillary porosity. Bulk density was determined by oven drying cores at 105 °C. Total porosity was calculated using 1 − bulk weight/soil particle density, assuming a soil particle density of 2.65 g/cm^3,36^. Disturbed soil samples were collected for determination of soil structure. Soil particle size was measured by Mastersizer 2000 laser particle analyzer^36^. Soil water contents at different soil suctions were determined by a high-speed refrigerated centrifuge (himac CR21G, Koki Holdings Co., Ltd., Japen) to evaluate wilting coefficient^3^.

Rain gauges were placed in the middle of the experimental site. In the Caragana shrubland with different Caragana densities of 87, 71, 51, 32 or 16 bushes per 100 m^2^, ten 100 m^2^ (5 m× 20 m) plots were prepared with 20-m sides constructed down the slope. Two 4-m long aluminum access tubes spaced 2 m apart were placed in the middle of each experimental plot. A neutron probe (CNC503A (DR), Beijing Nuclear Instrument Co., China) was used to monitor the field volumetric soil water content (VSWC) because of its high precision^37,38^. Before measuring VSWC, the neutron probe was calibrated for the study soil using standardmethods^12,39,40^. The calibration equation for this soil was *y* = 55.76*x* + 1.89, where *y* is VSWC and *x* is the ratio of the neutron count in the soil to the standard count^32^. The measuring depth ranges were 0–400 cm (2002–2006) and 0–860 cm (2011–2013), and measurements were conducted with 15-day intervals and 20-cm depth intervals. The VSWC obtained for each measuring depth was considered representative for the soil layer that included the measuring point ±10 cm depth, apart from that for the 5-cm depth, which was taken to represent the upper 10 cm of soil. The measurements included regular and irregular measurements: regular measurements were conducted in the growing season from mid-April to November for 2002 and to October for 2003–2006, and throughout the year for 2011–2014; irregular measurements were taken before and after each rainfall event.

The simulation experiment was conducted in nearby regions of the study site in the Caragana shrubland with a planting density of 87 shrubs per 100 m^2^ on 15 April 2002. A bucket of height 110 cm and diameter 80 cm was placed on the upper side of the slope, and the bucket was filled with water. The author used a representative Caragana shrub as a sample with mean height and crown width, and cut it at the base prior to the experiment. Nine aluminum access tubes were placed around the sample: one in the center near the Caragana stump after cutting the canopy and eight were installed at 50 and 100 cm away from the center in four directions because the planting density of caragana shrubs was approximately 100 cm × 100 cm. The experimental plot was located in the center of the simulation plot in order to express the mean MID. A soil ridge of 30 cm in diameter and 10 cm in height was created around the sample to prevent water flowing out from the infiltration area during the experiment. Water was transferred by a siphon of inner diameter of 4.7 mm from the bucket to the infiltration area and keep a thin water layer of 0.5–1.0 cm in the infiltration area. The rain record showed that the longest continuous rainfall was 5 days, the experiment ran for 8 days, commencing at 13:20 on 15 April and ending at 16:20 on 23 April 2002. We use subscript i to indicate measuring time. The measurements of VSWC with soil depth were made at time t_i_: t_i_ = 0 before the infiltration experiment and 1, 19, 26, 49, 74, 98, 122, 145 and 192 h after the simulated rainfall event.

### Statistical analysis

Soil water infiltration depth for different time intervals and soil water supply can be determined by analyzing neighboring soil moisture distribution curves with soil depth and soil water storage at the beginning and the end of a period. First, MID was analyzed in the Caragana shrubland with a Caragana density of 87 bushes per 100 m^2^, and then the effect of plant density on MID was analyzed. The significance of the soil water content measured in two tubes in the same plot on VSWC was analyzed using ANOVA with SPSS 13.0 software. Sample size is 28, which is soil water data measured in the 2002 and 2003.The p value of tube is 0.090, more than 0.05, and p value of the other factor variable are all 0, less than 0.05, which shows that the difference is not significant between tubes (two positions toward the sample shrub), however, soil water varies significantly among different densities, years and regions respectively. The difference of soil water content was not significant between two pipe positions because they are duplicate at the same soil depth and plot in the same year, and all other factors (i.e. plant density, year and soil depths) showed significant effects^13^. We used the average of data for the two pipes in the same plot or two core samples to express VSWC at the same soil depth and soil suction. Regression analysis was used to determine the relationships between VSWC and moisture suction using the least squares method. Data were transformed when necessary.

## Results

### Two-curve method for estimating infiltration depth

**Figure 2:**
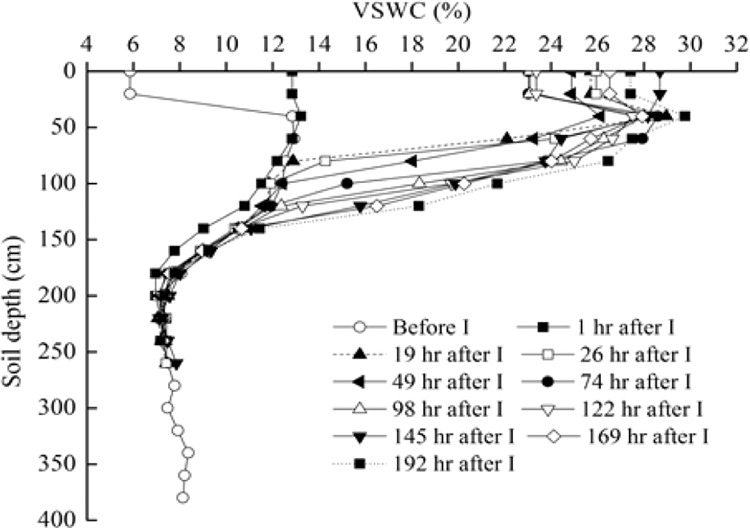
The dynamics of soil moisture with depth at different time (t) after infiltration experiment (I) at the center of infiltrating plot in the soil profile of Caragana shrubland. VSWC is volumetric soil water content.

The start and end of the infiltration process results in two respective soil water distribution curves for the soil profile. The changes in soil moisture content with time and depth during the simulated rainfall event are shown in Fig. 2.

Antecedent soil moisture was high in the 0–170 cm soil layer and low in the 170–390 cm layer in the shrubland. As infiltration progressed, a wetting front appeared and gradually deepened with time.

The VSWC in the deep soil layer immediately below the wetting front increased, suggesting that VSWC and infiltration depth increased with time, similar to results of Li *et al*.^25^. The soil water infiltration depth can be determined by the two-curve method using the soil moisture distribution curves at the beginning and the end of a period. As infiltration continued, the cumulative dynamic infiltration depth developed. The cumulative infiltration depths at different times after infiltration were 40 cm after 1 h, 80 cm after 19 h, 100 cm after 26 h, 120 cm after 74 h, 140 cm after 169 h and 160 cm after 192 h (Fig. 2). The maximum cumulative infiltration depth for the simulated experiment was 160 cm after 192 h. Thus, the MID was 170 cm.

### Two-curve method for estimating soil water supply

During infiltration, the water infiltrating into the soil surface (I_t_) can be partitioned into the increase in soil water storage of a unit volume of soil (S_t_), which is equal to soil water supply minus soil water evaporation; and the difference between inflow (F_1_) and outflow of downslope interflow (F_2_) in the unit volume of soil (Fs), which can be expressed as follows:

1 $${{\rm{I}}}_{{\rm{t}}}={{\rm{S}}}_{{\rm{t}}}+{{\rm{F}}}_{{\rm{s}}}$$

Where, S_t_ can be expressed as follows:

2 $${S}_{t}={\int }_{0}^{l}\theta (x,t)dx-{\int }_{0}^{l}\theta (x,0)dx$$

Where, the upper limit of the integral is the infiltration depth l for the rain event, and

3 $${{\rm{F}}}_{{\rm{s}}}={{\rm{F}}}_{2}\,\mbox{--}{{\rm{F}}}_{1}$$

The difference in outflow and inflow of downslope interflow in a unit volume of soil is very small and can be ignored because the soil was very porous, vertical structure was homogeneous, there was no less permeable soil layer in the profile, the slope was gentle and the VSWC was smaller than saturated water content during infiltration.

**Figure 3:**
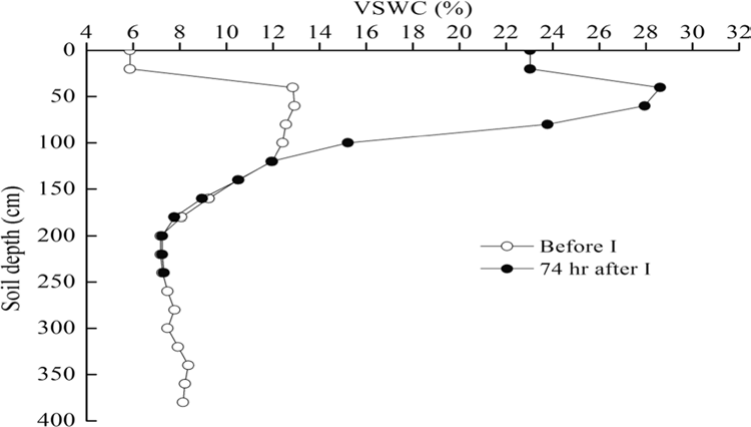
Changes in soil water contents with depth before and 1 hour after the infiltration experiment.

The soil water supply (mm) can be determined simply using the following formula:

4 $${{\rm{S}}}_{t}={\int }_{0}^{l}\theta (x,t)dx-{\int }_{0}^{l}\theta (x,0)dx={{\rm{W}}}_{2}-{{\rm{W}}}_{1}$$

where, St is in mm. During the infiltration experiment, water infiltrated into the soil from the surface and redistributed in the unsaturated zone. Using the data on the variation of soil moisture with depth and time in the soil profile, we determined the soil infiltration depths and soil water supply per rainfall event. Because the soil moisture profile distribution and its change with depth before and after a rainfall event are influenced by many factors, it is difficult to describe them using a simple formula in actual conditions. The soil water supply at a given time of the infiltration process can be considered as the difference between soil water storage in the infiltration depth after (W_2_) and before a given time (W_1_) (Fig. 3).

### Two-curve method for determining MID

The MID differed for the various distances in all directions. The maximum simulated infiltration depths were 130 cm at 50 and 100 cm in the east direction, at 50 cm in the south and at 50 cm in the west. The radius of the infiltration area was 15 cm, which was smaller than the 50 cm from the center of the simulated infiltration area and the loamy loess soil was porous, infiltration was mainly vertical and no interflow occurred in the soil profile.

No significant difference was found between data measured in the two tubes of the same plot. It was then possible to evaluate the infiltration depth and soil water supply for a rain event based on the two-curve method. One curve was the change of VSWC with depth before a rain event and the other was for after the rain event at the same location.

**Figure 4:**
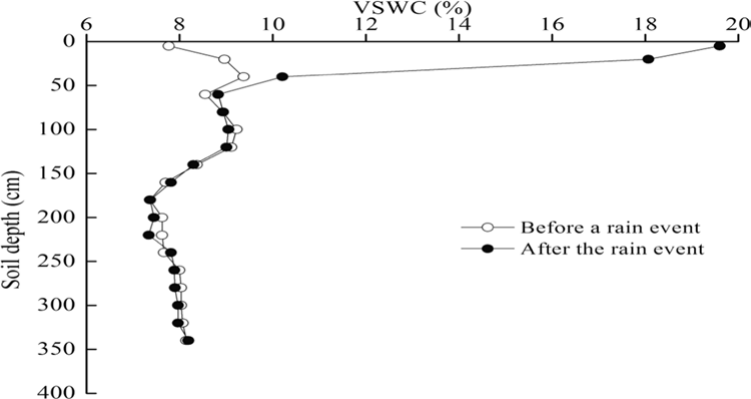
Changes in volumetric soil water content (VSWC) with soil depth before a rain event and after the rain event on 20 and 21 June 2002 in the soil under Caragana shrubland.

The weather forecast predicted a rainfall event on 20 June 2002. Thus, the distribution of soil moisture with depth before the rain event was monitored at 10 a.m. on 21 June 2002 and the distribution after the rain event was measured at 17:00 on 21 June 2002 (Fig. 4). The precipitation was 49.5 mm and a crossover point occurred at soil depth of 60 cm, representing an infiltration depth of 70 cm and soil water supply of 42.4 mm.

Rainfall is a discrete process and a rainfall event is the time interval between the occurrences of a period equal or larger in duration to a specific threshold: the minimum interval time (MIT)^41^. The MIT was 30 min in the study. After a rainfall event, soil water redistributes under the effect of gravity, soil matric suction and soil porosity.

**Figure 5:**
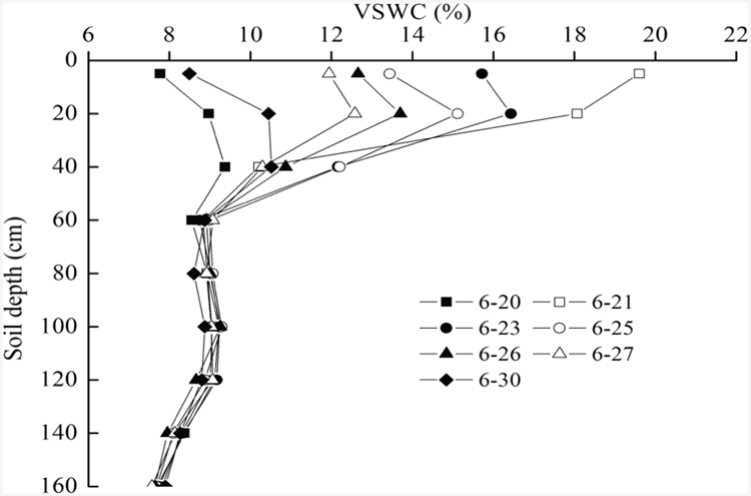
Changes in infiltration depths with time after three rainfall events: 49.5 mm on 20–21, 2.2 mm on 22 and 2.1 mm on 27 June, 2002 in Caragana shrubland. The minimum interval time of a rainfall event is 30 min. VSWC is volumetric soil water content.

The dynamics of soil moisture varied with time before and after three rainfall events during 20–30 June 2002 and the soil water moved down and the infiltration depth increased (Fig. 5). A higher water soil layer (HWSL_1_) was visible, which formed in the soil profile after the heavier rainfall event.

There were two forms of soil water movement in this HWSL_1_: moving to the rhizosphere or to surface soil due to plant transpiration and soil evaporation; and downward movement next to the wetting front due to matric suction or capillary head^41^, known as cumulative infiltration^41,42^ or reinfiltration^41^. As time elapsed, the cumulative dynamic infiltration depth developed. After two weeks, another higher water soil layer (HWSL_2_), in which the VSWC was lower than that for HWSL_1_, developed in a deeper location, and cumulative infiltration depth increased gradually with time. After analyzing the data collected during 2002–2006 and 2011–2014, the MID of soil water appeared in 2004, following the wet year of 2003.

**Figure 6:**
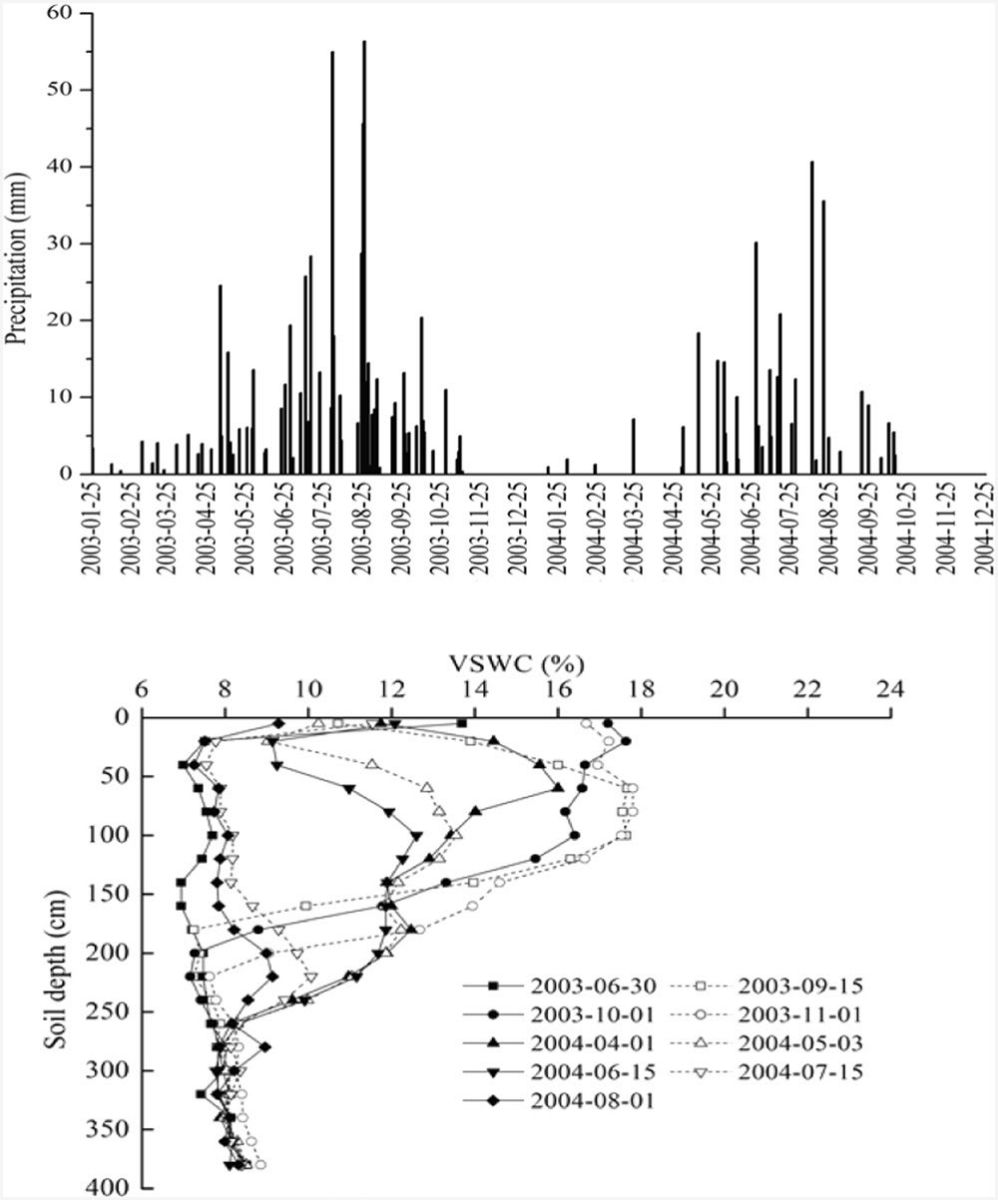
Daily change of precipitation during 2003–2004 (Figure above) and the Changes in soil infiltration depths with time in the period from 30 Jun 2003 to 1 August 2004 under artificial Caragana brush (Figure below).

The precipitation at the experiment station in 2003 was 623.3 mm, which was close to the maximum rainfall record of 634.7 mm in 1984. The daily change of rainfall from the beginning of year 2003 to the end of 2004 is shown in Fig. 6. There were continuous heavy rainfall events from late June to late August 2003 at the experiment site: 55.0 mm on 1, August, 45.7 mm on 25 August and 56.4 mm on 26 August 2003, respectively.

Soil water supply was lower than water evapotranspiration in June 2003, which resulted in a soil water deficit in Caragana shrubland with a density of 87 shrubs per 100 m^2^. Due to the effect of soil drought, Caragana leaves fell early in August and September 2003, and soil evaporation and plant root water uptake dropped sharply; the wetting front kept moving downward in the microenvironment due to the water potential gradient between the higher water soil layer at the wetting front and the dry soil layer below and next to the wetting front (Fig. 6). Finally, the cumulative infiltration depth increased gradually.

By 11 November 2003, the MID in 2003 was 210 cm. By 1 April 2004, the MID was 250 cm. After a period of fluctuation, the wetting front moved to 270 cm on 13 May 2004, and the VSWC at the wetting front fell below 9%. Although soil moisture at the wetting front was quite low and near the wilting coefficient, after one and a half months, the wetting front continued to slowly move down, and the maximum cumulative infiltration depth reached 290 cm by 1 August 2004. This implies that on a larger time scale, the maximum cumulative infiltration depth formed. During the experiment, the MID appeared in the year following the wettest year even though rainfall in 2004 was only 328.3 mm, which is far lower than the average rainfall of 415.6 mm in the region.

The MID differed according to density of Caragana shrubland, being 250, 270 and 290 cm for densities of 16 and 32, 51 and 71, and 87 shrubs per 100 m^2^, respectively. This suggests that density influenced the MID by reducing canopy interception and soil evaporation.

### The change in soil infiltration depth and soil water supply with precipitation

The relationship between the infiltration depth or soil water supply for different rainfall events and precipitation in the experimental area of Caragana shrubland with a density of 87 shrubs per 100 m^2^ is shown in Table 1. With increasing precipitation, the infiltration depth for one rain event and soil water supply subsequently increased. Regression analysis showed that the relationships between infiltration depth, soil water supply and precipitation in artificial Caragana shrubland could be expressed as:

5 $${\rm{ID}}=0.7098{\rm{P}}+32.454,\,{{\rm{R}}}^{2}=0.9919$$

6 $${\rm{SWS}}=0.6984{\rm{P}}+2.1336,\,{{\rm{R}}}^{2}=0.9919$$

Where, ID is infiltration depth for one rain event (cm), SWS is soil water supply (mm) and P is precipitation (mm). The relationship will change with soil, vegetation type and region.

**Table 1:** Infiltration depth and soil water supply under different rainfalls.

Date	Precipitation (mm)	Infiltration depth (cm)	Soil water supply (mm)
29 June 2003	19.4	50	16.9
15 July 2003	28.4	50	12.8
5–6 May 2003	29.6	50	26.1
20–21 June 2002	49.5	70	42.4
21–26 August 2003	137.6	130	97.2

## Discussion

The results measured in the laboratory cannot be applied to field conditions^3,31,32^. The flume infiltrometer and simulated rainfall cannot be used to measure soil infiltration depth. Electrode measurements can be used to measure water circulation in the soil in terms of direction and the amount of flow at different depths in the soil profile^21^. It is difficult to determine MID for simulated rainfall. Determining MID by the oven drying method is difficult to estimate the infiltration depth.

In Huangmian soil, the downslope interflow could be ignored because the soil vertical structure was almost homogeneous, there was no impermeable soil layer in the profile, the VSWC was less than saturated water content when infiltrating, the slope was gentle and the difference between outflow and inflow of downslope interflow in a unit volume of soil was less than the error range. In a long-term study, we should determine the start time to measure soil water after the rainfall event because it influences the soil water supply and infiltration depth for this event. If the difference in outflow and inflow of downslope interflow in a unit volume of soil exceeded the error range, it could not be ignored, and soil water supply per rainfall event was added to the difference between outflow and inflow of downslope interflow. The MID is the deepest point of cumulative infiltration depth in a long-term. This method is more suitable for use in deeper soils, such as most soils of the Loess Plateau. Reducing the measurement depth intervals from 20 to 10 or 5 cm, thus reducing maximum error from 20 to 10 or 5 cm, or using a sensor to measure the VSWC at different soil depth, would improve measurement precision.

The maximum cumulative infiltration depth of the simulated experiment was 170 cm and smaller than 290 cm. The maximum cumulative infiltration depth of 290 cm was within the range of 100–300 cm^10^, which approved that the estimating method is right.

Under natural conditions, the two-curve method also can be used to determine the use depth of soil water and soil water consumption by plants in a given period.

## Conclusions

Infiltration depth and soil water supply for one rain event could be estimated by the two-curve method, and MID could be estimated by a series of two-curve methods. The infiltration depth for one rain event was equal to the distance from the surface to the crossover point between the two soil water distribution curves with soil depth before a rain event and after the rain event. The soil water supply for one rainfall event was the difference of the soil water resources in the soil layers of the infiltration depth before and after the rain event, and maximum infiltration depth that occurred after a long period, and could be estimated by a series of two-curve methods. A maximum infiltration depth of 2.9 m occurred in the artificial Caragana shrubland and appeared in the year following the wettest year. The relationship between infiltration depth or soil water supply and the precipitation outside of the artificial Caragana shrubland can be expressed in a linear equation. Plant density influenced both MID and soil water supply. This discovery is very important for estimating SWRULP and SWCCV and regulating the relationship between soil water and plant growth.
